# The Impact of the Natural, Social, Built, and Policy Environments on Breast Cancer

**DOI:** 10.15436/2378-6841.15.020

**Published:** 2015-08-03

**Authors:** Steven S. Coughlin, Selina A. Smith

**Affiliations:** 1 Department of Health Science and Sustainability, Division of Public Health, University of Massachusetts, Lowell, MA; 2 Institute of Public and Preventive Health, and Department of Family Medicine, Medical College of Georgia, Georgia Regents University, Augusta, GA

**Keywords:** Breast cancer, DDT, Dioxins, Environment, Obesity, PCBs, Physical activity, Policy

## Abstract

**Background:**

The global burden of breast cancer in women is substantial and increasing. Efforts to address breast cancer have focused on primary prevention, reduction of modifiable risk factors, early detection, timely referral for appropriate treatment, and survivorship. Environmental and lifestyle factors that increase breast cancer risk include ionizing radiation, exogenous hormones, certain female reproductive factors, alcohol and other dietary factors, obesity, and physical inactivity. A variety of chemical exposures are purported to be associated with breast cancer.

**Methods:**

In this article, we summarize the influence of the natural, social, built, and policy environments on breast cancer incidence and cancer recurrence in women based upon bibliographic searches and relevant search terms.

**Results:**

Despite a lack of conclusive evidence from epidemiologic studies, exposures to chemicals with estrogenic or other properties relevant to sex steroid activity could influence breast cancer risk if the exposures occur at critical life stages or in combination with exposure to other similar chemicals. Results from several studies support an association between shift work and disruption of the circadian rhythm with breast cancer risk. The social environment likely influences breast cancer risk through several mechanisms including social norms pertaining to breast feeding, age at first live birth, parity, use of oral contraceptives and replacement estrogens, diet, and consumption of alcohol. Social norms also influence body weight, obesity, and physical activity, which have an effect on risk of breast cancer incidence and recurrence. Obesity, which is influenced by the social, built, and policy environments, is a risk factor for the development of postmenopausal breast cancer and certain other cancer types.

**Conclusions:**

The natural, social, built, and policy environments affect breast cancer incidence and survival. Effective health care policies can encourage the provision of high-quality screening and treatment for breast cancer and public education about the value of proper diet, weight control, screening and treatment. Additional research and policy development is needed to determine the value of limiting exposures to potentially carcinogenic chemicals on breast cancer prevention.

## Introduction

Breast cancer is a leading cause of morbidity and mortality among women in the U.S. and many other countries^[1]^. The global burden of breast cancer in women, measured by incidence, mortality, and economic costs, is substantial and increasing^[2]^. Efforts to alleviate this burden have focused on primary prevention and modifiable risk factor reduction, on early detection and timely referral for appropriate treatment, and on breast cancer survivorship issues. Various factors, genetic and environmental, or the interaction between the two, increase the risk of breast cancer incidence and recurrence^[1]^. Environmental and lifestyle factors that increase breast cancer risk include ionizing radiation, exogenous hormones, certain female reproductive factors, alcohol and other dietary factors, obesity, and physical inactivity. The increasing prevalence of obesity in the U.S. and many other countries and the independent association of obesity with cancer incidence have prompted interest in identifying environmental influences on risk of obesity and breast cancer^[3]^. Obesity, a risk factor for the development of postmenopausal breast cancer and certain other cancer types, is associated with poorer response to cancer therapy and cancer reoccurrence^[4]^.

Among women who have already been diagnosed with breast cancer, obesity is associated with breast cancer recurrence and poorer survival^[5]^. Maintaining a healthy body weight reduces a woman’s risk of cancer recurrence, diabetes and cardiovascular diseases^[6]^. Breast cancer-related environmental factors, however, extend beyond individual exposures to include the natural, built, social, and policy environments^[7]^.

In this article, we reflect on the influence of natural, social, built, and policy environments on breast cancer incidence and cancer recurrence in women, discuss remaining challenges in this area, and offer suggestions for additional research and policy development. As described by Juarez et al. (2014), the natural, built, social, and policy environments play important roles in health and disease including racial and ethnic disparities in breast cancer.

## Materials and Methods

Our review is based upon bibliographic searches in PubMed and Google Scholar and relevant search terms. For example, we identified articles published in English in recent years using the following MeSH search terms and Boolean algebra commands: (((diet weight or dietary or diet weight loss or dietary intake or diet cancer or nutritional or health nutrition or cancer nutrition or cancer nutrition) or (weight loss or weight gain or body weight or exercise weight or weight management)) and women). We also identified articles using the following combination of MeSH search terms: ((breast cancer and (environmental factors or environmental risk factors or environmental exposure or environmental pollution or organochlorines or polychlorinated biphenyls or dichlorodiphyenyl-trichloroethane)). The searches were not limited to words appearing in the title of an article. Information obtained from bibliographic searchers (title and topic of article, information in abstract, geographic locality of a study, and key words) was used to determine whether to retain each article identified in this way. In addition, we identified reviews included in Cochrane reviews (http://community.cochrane.org/cochrane-reviews) and the U.S. Institute of Medicine^[8]^ and reviewed the references of reports and review articles.

## Results

### Breast Cancer and the Natural Environment

The natural environment includes physical, chemical, and biological factors. Here we consider the natural environment broadly to include synthetic chemicals that occur in nature due to human activities. Exposure to ionizing radiation, which can induce mutations in DNA is an established breast cancer carcinogen^[9,10]^. A variety of chemical exposures have been purported to be associated with breast cancer. The U.S. Institute of Medicine^[8]^, however, concluded that the evidence associating individual chemicals with breast cancer risk is not conclusive and recognized the need for further research in this area. The IOM noted that exposure to chemicals with estrogenic or other properties relevant to sex steroid activity, such as bisphenol A, polybrominated diphenyl ethers, and certain dioxins or dioxin-like compounds, may influence breast cancer risk. Several epidemiologic studies have found associations between occupational exposes to solvents and breast cancer in women, but the causality of the associations is uncertain^[11–13]^. In population-based epidemiologic studies, organochlorines, a diverse group of synthetic chemicals that include polychlorinated biphenyls (PCBs), dioxins, and pesticides such as dichlorodiphenyl-trichloroethane (DDT), lindane and hexachlorobenezene, have not been consistently associated with breast cancer risk^[14–16]^. Although use of DDT and PCBs has been banned in the United States since the 1970s, organochlorine compounds have accumulated and persisted within the environment, and some are still used in some low- or middle-income countries. As a result, measurable amounts of these chemicals can be found in human tissues. Because some organochlorine compounds are estrogen agonists or antagonists, as determined in cell culture and animal studies, a possible link between breast cancer risk and organochlorine exposure has been hypothesized. Although results from some studies support this hypothesis, most epidemiological studies do not^[15]^. While these compounds may have other adverse environmental or health effects, organochlorine exposure is not believed to be causally related to breast cancer. In the California Teachers Study^[16]^, no associations were found between residential exposure to ambient estrogen disruptors and overall breast cancer risk or hormone receptor-positive breast cancer risk, or among targeted subgroups of participants (pre-/peri-menopausal women, post-menopausal women, never-smokers, non-movers, and never-smoking non-movers). Among never-smoking non-movers, however, elevated risks for hormone receptor-negative tumors were noted for higher exposure to cadmium compounds and possibly to inorganic arsenic^[16]^. The risk of breast cancer from exposure to 2,3,7,8-tetrachlorodibenzo-p-dioxin (TCDD) has been reviewed by various authors and expert panels who found no consistent evidence of an increased risk^[17]^. In a recent prospective study in France, no association was found between estimated dietary dioxin exposure and breast cancer^[18]^. Despite the lack of conclusive evidence from epidemiologic studies, exposures to chemicals with estrogenic or other properties relevant to sex steroid activity could influence breast cancer risk if the exposures occur at critical life stages or in combination with exposure to other similar chemicals^[8,17]^. Results from several studies support an association between shift work and disruption of the circadian rhythm with breast cancer risk. Levels of serum melatonin, which may have a protective effect, decrease when people are exposed to light at night^[19]^.

### Breast cancer and the Social Environment

The social environment likely influences breast cancer risk through mechanisms that include social norms pertaining to breast feeding, age at first live birth, parity, use of oral contraceptives and replacement estrogens, diet, and consumption of alcohol^[20]^. Social norms also influence body weight, obesity, and physical activity, which in turn influence risk of breast cancer incidence or recurrence^[21]^. Other aspects of the social environment that affect breast cancer risk include cultural beliefs and attitudes (e.g., fatalism) and the availability of social networks and community organizations and agencies that help provide access to information and resources for breast cancer prevention, early detection, treatment, and survivorship. In many traditional cultures, including parts of the Middle East, Africa, and Asia, efforts are underway to combat cultural attitudes that deter women from talking about breast cancer or seeking care^[2]^.

### The Impact of the Built Environment

Affecting breast cancer are several aspects of the built environment including the availability of transportation to and from clinics and hospitals that provide screening and treatment services. The location of diagnostic and treatment facilities outside of low-income, minority neighborhoods can also pose a barrier to receiving health care services. Several studies have examined commuting time in relation to receipt of breast cancer screening services^[22]^. Proximity to recreational facilities, bike lanes, walking and running paths, and stores and markets that offer nutritious and affordable foods (e.g., fresh fruits and vegetables) can contribute to reduction in risk of chronic disease^[23,24]^. In addition, population studies have identified neighborhood effects and aspects of the built environment that may influence breast cancer incidence and mortality^[25,26]^.

### Breast Cancer and the Policy Environment

Health policies at the national, state or provincial, and local level impact breast cancer incidence and survival. Effective health care policies can encourage the provision of high-quality screening and treatment for breast cancer and public education about the value of proper diet, weight control, screening and treatment. Organized approaches for delivering breast cancer screening should be accompanied by programs and policies that provide access to timely and appropriate diagnostic follow-up and treatment services. In many countries, large numbers of women lack access to screening mammography services or to high-quality oncology care. Public policies aimed at providing equitable health care coverage and addressing inequities in socioeconomic status and health can contribute to alleviating the burden of breast cancer and other serious illnesses.

Public policies at the community level and in work-places can encourage development of health promotion strategies, including those that promote physical activity (for example, recreational facilities, bike lanes, and paths for walking and running) or eating a healthy diet^[23,24,27–30]^.

The policy environment also determines permissible exposures to endocrine-disrupting chemicals that may be linked to breast cancer but for which the evidence from epidemiologic studies is inconclusive. The U.S. Environmental Protection Agency estimates that there are 10,000 endocrine-disrupting chemicals among the common daily exposures that could affect risk of disease^[31]^. Expert panels have begun to address the daunting tasks of identifying, characterizing, and elucidating the mechanisms of endocrine- disrupting chemicals in breast cancer in order to produce a comprehensive model that will facilitate preventive strategies and public policy^[31]^.

## Discussions

The natural, social, built, and policy environments affect breast cancer incidence and survival. Effective health care policies can encourage the provision of high-quality screening; treatment for breast cancer; and public education about the value of diet, weight control, screening, and treatment. It is likely that, in addition to genetic and biological factors, a variety of social, structural, and environmental factors influence breast cancer incidence and survival^[1,21]^. Results from epidemiologic studies indicate that greater body fatness and alcohol consumption are associated with a higher risk of several chronic illnesses including postmenopausal breast cancer^[32]^. According to the American Institute on Cancer Research (AICR), eating a healthy diet, maintaining a healthy weight, and being physically active can prevent about one-third of the most common cancers in the U.S^[30]^. To reduce risk of recurrence, the AICR recommends that cancer survivors adhere to cancer prevention guidelines.

## Conclusion

Despite a lack of conclusive evidence from epidemiologic studies, exposures to chemicals with estrogenic or other properties relevant to sex steroid activity could influence breast cancer risk if the exposures occur at critical life stages or in combination with exposure to other similar chemicals. Results from several studies support an association between shift work and disruption of the circadian rhythm with breast cancer risk. Additional research and policy development are needed to assess the value of limiting exposures to potentially carcinogenic chemicals for breast cancer prevention. Further, synthetic chemicals that have not been causally linked to breast cancer may pose hazards for the development of other chronic illnesses and adverse reproductive health outcomes.
